# Application of the Industrial Byproduct Gypsum in Building Materials: A Review

**DOI:** 10.3390/ma17081837

**Published:** 2024-04-16

**Authors:** Zhiqing Xie, Xiaoming Liu, Zengqi Zhang, Chao Wei, Jiarui Gu

**Affiliations:** 1 School of Metallurgical and Ecological Engineering, University of Science and Technology Beijing, Beijing 100083, China; xiezhiqing8023@163.com (Z.X.); weichao0810@126.com (C.W.); gjr18832313078@126.com (J.G.); 2 State Key Laboratory of Advanced Metallurgy, School of Metallurgical and Ecological Engineering, University of Science and Technology Beijing, Beijing 100083, China

**Keywords:** byproduct gypsum, building materials, environmental problems

## Abstract

The industrial byproduct gypsum is a general term for byproducts discharged from industrial production with calcium sulfate as the main ingredient. Due to the high number of impurities and production volume, the industrial byproduct gypsum is underutilized, leading to serious environmental problems. At present, only desulfurization gypsum and phosphogypsum have been partially utilized in cementitious materials, cement retarders, etc., while the prospects for the utilization of other byproduct gypsums remain worrying. This paper mainly focuses on the sources and physicochemical properties of various types of gypsum byproducts and summarizes the application scenarios of various gypsums in construction materials. Finally, some suggestions are proposed to solve the problem of the industrial byproduct gypsum. This review is informative for solving the environmental problems caused by gypsum accumulation.

## 1. Introduction

The industrial byproduct gypsum refers to the byproduct or waste residue generated by chemical reactions in industrial production with calcium sulfate as the main component. It is also known as chemical gypsum or industrial waste gypsum, and the main component is calcium sulfate dihydrate (CaSO_4_·2H_2_O) [1]. According to the output industry and species, the industrial byproduct gypsum mainly includes desulfurization gypsum, phosphogypsum, titanium gypsum, citrate gypsum, fluorogypsum, and salt gypsum.

**Figure 1:**
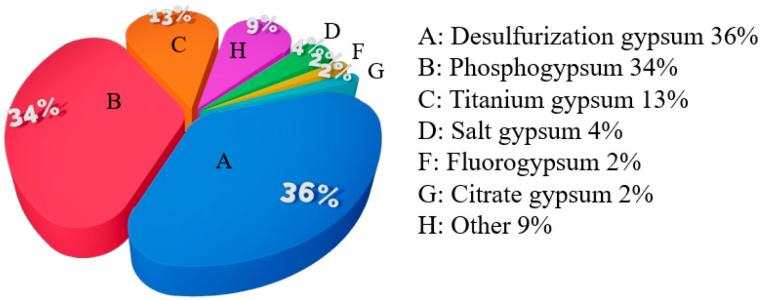
Main sources of the industrial byproduct gypsum in China [2,3].

At present, the cumulative stock of the industrial byproduct gypsum in China exceeds 1100 Mt. In 2020, the total production of the industrial byproduct gypsum in China was approximately 200 Mt [2], of which the production of desulfurization gypsum, phosphogypsum, titanium gypsum, and salt gypsum accounted for more than 90%, as show in Figure 1 [3]. A large amount of gypsum byproduct cannot be utilized, leading to massive stockpiling and land occupation, with the risk of polluting water bodies and soil. The treatment of the industrial byproduct gypsum has become a challenge for the development of various industries [4,5].

According to statistics, China’s annual industrial byproduct gypsum emissions are approximately 280 million tons, with a utilization of approximately 142 million tons and an overall comprehensive utilization rate of approximately 51%. For example, the comprehensive utilization rate of phosphogypsum has increased from 20% to 45% in the past ten years. Many enterprises and scientific research institutions have performed much research and practical applications in the field of comprehensive phosphogypsum utilization. Various types of research have yielded results in the fields of architecture, agriculture, medicine, etc., with the most extensive applications in building materials [6,7,8].

**Figure 2:**
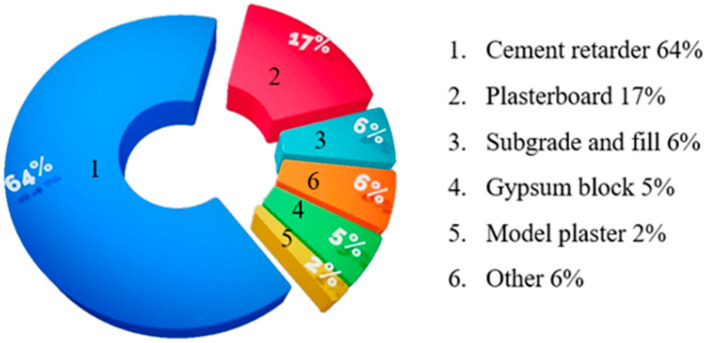
Main gypsum consumption areas in China in 2020 [2,12].

In the building materials, the industrial byproduct gypsum is mainly used in the production of cement retarders, gypsum slats, gypsum bricks, gypsum blocks, etc. In recent years, new gypsum products, such as self-leveling gypsum, α-type high-strength gypsum, ii-type anhydrous gypsum, and calcium sulfate whiskers, have also been developed [9,10]. In addition, the industrial byproduct gypsum is used in road construction, filling and other applications [2,11]. The main gypsum consumption areas in China in 2020 are shown in Figure 2.

However, the complex and variable composition of the industrial byproduct gypsum seriously hampers its resource utilization. To increase the application of the industrial byproduct gypsum, it is necessary to develop low-cost and efficient decontamination and purification processes. Moreover, the application fields of gypsum byproducts, such as cementitious materials, road base materials, and mine filling materials, should be expanded, especially in the direction of larger application amounts [13,14].

This paper reviews the application of six industrial gypsum byproducts in the building materials industry. The various methods of application are summarized and analyzed. In addition, their advantages and improvements are discussed. Finally, a treatment for the industrial byproduct gypsum is proposed, and some suggestions are given. It can be helpful for researchers in the future to realize the disposal and utilization of the industrial byproduct gypsum.

## 2. Overview of the Industrial Byproduct Gypsum

### 2.1. Source

Desulfurization gypsum (DG) is the byproduct of the limestone–gypsum flue gas desulfurization process in coal-fired power plants [15]. In China, the annual production of DG can reach up to 80 million tons, and the utilization rate has been around 80% in recent years. However, the comprehensive utilization rate has been reported to be close to 100% in countries like Japan, Germany, the United States, and the United Kingdom [16]. Among them, Japan and Germany are noted for having the most advanced technology in this field. In Germany, the production of DG has reached 5~6 Mt/a, with an average utilization rate exceeding 97.5%. This material is primarily utilized in the manufacturing of paper gypsum board and cement additives. Additionally, it is used in the production of gypsum slag board, binder, building gypsum, roadbed, and for land leveling, which necessitates sand and other soot materials [17].

The primary method for producing high concentrations of phosphoric acid from sulfuric acid and phosphate rock is the wet process. This process results in the generation of a significant quantity of solid phosphogypsum (PG) waste [18]. According to the statistics, the wet process for phosphoric acid production generates 5 tons of PG for every 1 ton of phosphoric acid (P_2_O_5_) produced [19]. The global cumulative emissions of PG amount to around 6 billion tons. Presently, only 40% of PG resources are being utilized [20]. Given that PG contains various detrimental impurities, such as phosphorus and fluorine, its presence significantly affects the environment and water [21]. Presently, the extensive utilization of PG in the primary PG-producing nations worldwide predominantly takes place in the agricultural, construction, and road sectors. The utilization focus varies based on the unique conditions present in each country [22]. For instance, the predominant utilization of PG involves storage and processing, with a minor portion allocated to agricultural purposes in Brazil, Finland, and United States. Conversely, in Belgium, India, and Japan, PG is used primarily in cement production. Additionally, in Russia and the Philippines, PG is instrumental in road construction and the recycling of rare earth elements [23].

There is also some underutilized gypsum. Titanium gypsum (TG) is a solid waste generated during the preparation of titanium dioxide by the sulfuric acid method [24], and the storage of TG is gradually becoming an environmental problem [25]. Fluorogypsum (FG) [26] is an industrial byproduct produced in the fluoride salt industry when preparing hydrofluoric acid. Since FG is composed of anhydrous gypsum, it hydrates slowly and does not have early strength, so it is difficult to develop and utilize it directly [27].

### 2.2. Characteristics

The industrial byproduct gypsum particles are fine, and the particle size distribution range is small, generally ranging from 20 to 60 μm, with uneven gradation. Compared with those of natural gypsum, the crystal growth of gypsum is poor, and the influence of impurities leads to poor mechanical and rheological properties in gypsum [6]. Table 1 shows the physical properties of desulfurization gypsum (DG), phosphogypsum (PG), titanium gypsum (TG), and fluogypsum (FG).

As the content of CaSO_4_·2H_2_O in the industrial byproduct gypsum usually reaches 70%, its CaO and SO_3_ contents are more than 70%, and the remainder contains a small amount of SiO_2_ and Al_2_O_3_ [1,28]. Various forms of gypsum may contain distinct impurities. A significant quantity of Fe_2_O_3_ is found in TG, while the content of CaSO_4_ is relatively low [29]. PG comprises minor quantities of H_3_PO_4_, Ca(H_2_PO_4_)_2_·H_2_O, CaHPO_4_·2H_2_O, and various trace metals [30]. FG contains CaF_2_ and H_2_SO_4_ in the range of 1% to 3% [31]. Table 2 shows the chemical composition of some of the gypsum byproducts.

**Table 1:** Physical properties of various types of gypsum.

Species	Density (g/cm^3^)	Water Content (%)	Specific Surface Area (m^2^/kg)	Mean Particle Size (μm)	pH	Refs.
DG	1.06–1.30	10–15	150–230	30–60	6–9	[1,6,29]
PG	1.05–1.20	25–35	450–520	5–150	1–4.5	[30,32]
TG	2.00–2.50	30–65	780–820	15–60	6–9	[33,34]
FG	1.30–1.50	15–20	300–500	10–80	2–4	[31,35,36]

## 3. Application in Building Materials

Around the world, a large amount of the industrial byproduct gypsum is generated annually. Moreover, the accumulation of gypsum is increasing annually. The industrial byproduct gypsum has been extensively studied in building materials. Its applications in different materials are listed in Table 3.

### 3.1. Cementitious Material

After a series of physical and chemical effects, building materials can change from slurry to solid stone. They cement other solid materials into a whole material and have a certain mechanical strength, and are collectively referred to as cementing materials.

**Figure 3:**
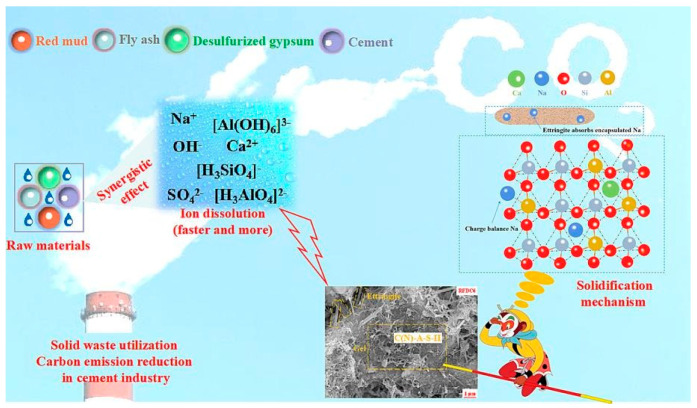
Hydration mechanism diagram [40].

DG, as a raw material for building material production, produces no discharge of waste residue or waste water during recycling and utilization [17], and has been widely used in cementitious materials [52]. DG, a sulfate solid waste, reacts rapidly with alkaline solid waste in combination with reactive silica–alumina solid waste [40]. Cementitious materials were prepared using red mud, fly ash, and DG. In the macroscopic experiment, it was found that the strength of the cementitious material reached a maximum when the yield of desulfurized gypsum was 6%. Figure 3 shows that SO_4_^2−^ in DG can react with Ca^2+^ in C-S-H or C (N) -A-S-H gels to replace [SiO_4_]. The displaced [SiO_4_] can react with free Ca^2+^ to form a new gel, and the presence of [SiO_4_] will also increase the solubility of the active Al. Moreover, the synergistic effect of the three solid wastes results in curing Na^+^, and calcite has the ability to absorb and encapsulate Na^+^ [34]. The positive valence state of sodium ions can be balanced by the charge of alumina tetrahedra with a negative valence, and chemical bonds are formed between sodium and aluminum atoms to achieve stable curing of sodium ions [31].

DGs are more commonly used in cementitious materials, but most of them are used as retarders, and the dosage is small, generally less than 10%. Anne Thymotie studied the effect of DG on fly ash cementitious materials [53]. The DG was pretreated at 150 °C to transform the gypsum dihydrate in the DG to gypsum hemihydrate, which was then used to replace the fly ash with 3%, 5%, and 10%, respectively. The results showed that the compressive strength and thermal conductivity of the cementitious materials increased with increasing DG. Meanwhile, higher DG enhances the late strength of the cementitious materials, mainly because the addition of DG increases the sulfate in the system, which in turn enhances the amount of ettringite. When the dose of DG is too high, the setting time of cementitious materials can be greatly extended; therefore, if a large amount of DG is used, the problem of excessive retardation must be solved. Using DG, slag powder, and steel slag as auxiliary materials [54], supersulfurous, water-hardening cementitious materials were prepared, in which the DG dose was greater than 40%, and when only DG and slag powder were used as raw materials, the strength of the prepared materials partially extended the standard, although the strength partially exceeded the standard. When only DG and slag powder, as well as a small amount of clinker as a raw material, are used, although the prepared cementitious materials are able to meet the standard, the initial setting time reaches 11 h, while a small amount of steel slag instead of clinker is conducive to the stable growth of calcium alumina. And the initial setting time drops to 7 h. Supaporn Wansom studied the performance of cementitious materials prepared from 20% DG, 40% fly ash, and 40% cement in order to improve the utilization of DG [55]. It was found that the compressive strength of the material was maximized at this ratio, and it had high water resistance. The generation of hydration products was found to cover the gypsum crystals and reduce the dissolution of gypsum through microscopic experiments, thus improving the durability properties of the materials. At present, a large amount of DG can only be used for part of the special cementitious materials in general cement silicate cement, and the amount of sulfur–aluminate cement in the mixture is still relatively small.

**Figure 4:**
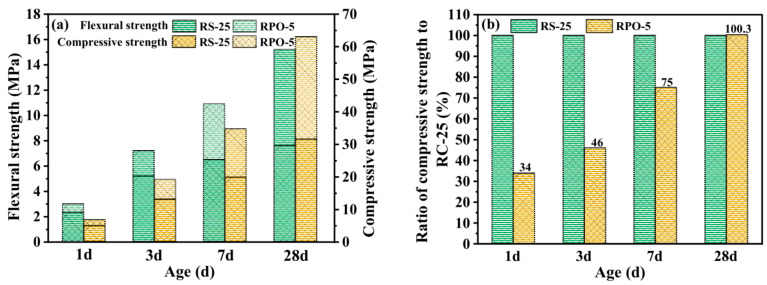
Mechanical properties of the samples containing RS-PG (**a**) compressive strength compared to that of RS-25 (**b**) [46].

PG can enhance red sandstone volcanic ash activity to produce red sandstone–PG–cement composites (RS-PG-OPCs) [46]. Experiments have shown that red cement blended with red sandstone (RS) improves the fluidity of the slurry, but decreases its mechanical strength. Experiments have shown that this is because the active SiO_2_ in the raw material can react with calcium hydroxide to form a gel, but only a small amount of calcium hydroxide is utilized, and RS is found to have volcanic ash activity. In addition, after the addition of 5% PG, the hydration process of the former is slowed down (RPO-5), the compressive strength of the sample decreases gradually with age, and the compressive strength at 28 days is at the same level as that of RS-25 in Figure 4. In addition, harmful ions in PG can reduce the performance of the sample and slow down the hydration reaction.

PG has been used in several studies on gelling materials. Only PG and cement have been used to prepare cementitious materials to study the effect of PG doping on the properties of cementitious materials [30]. PG content affected the rate of hydration reactions. At the same time, the greater the PG doping was, the greater the porosity, but the pore size distribution of the mortar improved to a small extent when 10% PG was doped compared with when no PG was doped. The mechanical properties of the mortar specimen exhibited the same pattern: the 28 d compressive strength first increased and then decreased, and reached the maximum value when the PG doping was 10%, which was 4.98 MPa higher than the strength of cement. The compressive strength of mortar can meet the national standard when the PG doping is less than 30%. This is mainly because PG can participate in secondary reactions to generate calcium alumina, and the microstructure is more compact. Using PG to replace part of the cement realizes the resourceful use of the industrial byproduct gypsum. Girts Bumanis studied a ternary system of cementitious materials to prepare high-performance cementitious materials from PG, cement, and volcanic ash [56]. The PG content was up to 50% and the compressive strength of the cementitious material was more than 50 MPa, while the smoke study showed that the lightweight foam concrete prepared by the ternary system also has good mechanical properties, which provides a new way to utilize this kind of solid waste material. Aziz Azifa utilized 5% PG to improve the mechanical properties of cement clinkers [57]. It was found that the optimum water–cement ratio for mortar is 0.35, and an excessive water–cement ratio reduces the mechanical properties of the material. A significant amount of C-S-H and ettringite generation was determined via a microanalysis. It was shown that a small amount of PG had a positive effect on the properties of cementitious materials.

A cementitious material was prepared by adding ordinary silicate cement, granulated blast furnace slag, sulfoaluminate clinker, and fly ash to TG, as the raw material [49]. The study showed that in the cementitious material system, the compressive strength increased due to the increase in PC doping; gypsum doping increased and decreased, and the strength decreased with the addition of exciters. The volume expansion decreased as the proportion of mineral increased. The longer the expansion time, the greater the development of the later volume expansion rate. At the same time, the volume of the cementitious material during 14 days of maintenance before rapid expansion and after 14 days tended to stabilize. The optimal proportion was 10% cement, 30% mineral powder, 5% sulfoaluminate clinker, 20% fly ash, and 35% TG, and the 28 d compressive strength of this proportion reached 34.1 MPa.

**Figure 5:**
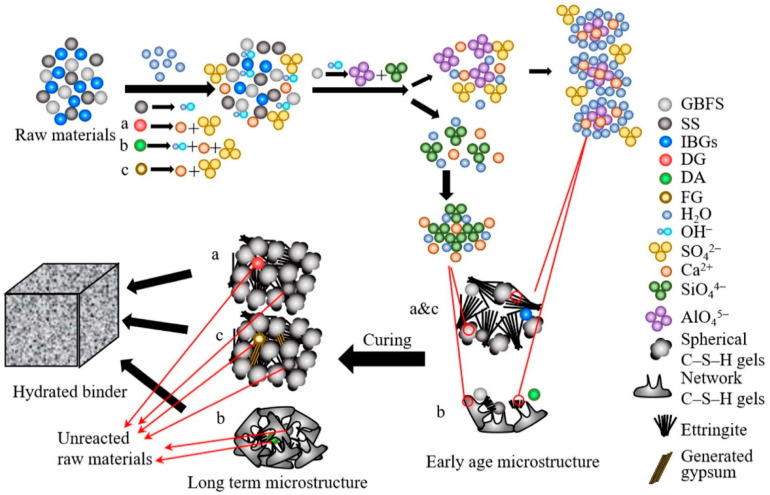
Activation mechanisms: (**a**) DG, (**b**) DA, and (**c**) FG [59].

FG-based cementitious materials include 40–45% FG, 50–55% granulated blast furnace slag, 5% cement, and 1% K_2_SO_4_ [35]. The paste strength of these materials increases with increasing hydration, and the content of FG should not be too high and should be less than 45%. Additionally, the performance of these materials should be greater than the strength index of 42.5-grade composites. Additionally, their performance is greater than the strength index of the 42.5-grade composite silicate cement (Chinese standard GB175-2007) [58], which is a suitable substitute for cement as a water–hard cementitious material. Chengwen Xu [57] investigated the strength and hydration mechanisms of three solid wastes, DG, desulfurization ash (DA), and FG, prepared with steel slag and granulated blast furnace slag, respectively. Figure 5 shows the activation mechanism of the three solid wastes on the steel slag (SS)-granulated blast furnace slag (GBFS)-based cementitious materials, while DG and FG provide Ca^2+^ and SO4^2−^ to produce C-S-H gel and acicular calomel, which form a reticulated structure to improve the strength. With time, the newly generated C-S-H gel fills the gaps between the acicular calcite layers, and the strength gradually increases. The addition of desulfurization ash resulted in a decrease in compressive strength in the early stage and an increase in compressive strength in the later stage. In the process of C-S-H gel generation, calcium hydroxide in DA promotes the dispersion of AlO_4_^5−^ and SiO_4_^4−^ in granulated blast furnace slag, which provides space for the C-S-H gel to generate a lattice structure. Therefore, all three solid wastes have a certain activation effect on slag-based, steel slag-granulated blast furnace gelling materials, providing alternative materials for producing green building materials.

The cementitious materials were prepared using the industrial byproducts gypsum, DG, PG, TG, FG, and SG. It can be seen from various studies that the dosage of DG in cementitious material is between 5% and 10%. The dosage of PG can reach 30%, and it has a great effect on the strength of the cementitious material when it is more than 30%. The dosage of TG can reach 35%, and it can also reach good strength under the activation of the activity with the fly ash. The amount of FG used in the preparation of cementitious material with granulated blast furnace slag and cement is as high as 40%, and the strength of the material remains constant. At present, DG is basically used as a supplementary material in cementitious materials, while PG, TG, and FG have been studied as main materials, and the dosage is up to 30%.

### 3.2. Cement Retarder

Cement retarders are admixtures that can delay the hydration reaction of cement, thus prolonging the setting time of concrete, maintaining the plasticity of fresh concrete for a long time, facilitating casting, and improving construction efficiency; at the same time, it has no adverse effect on the performance of the material.

**Figure 6:**
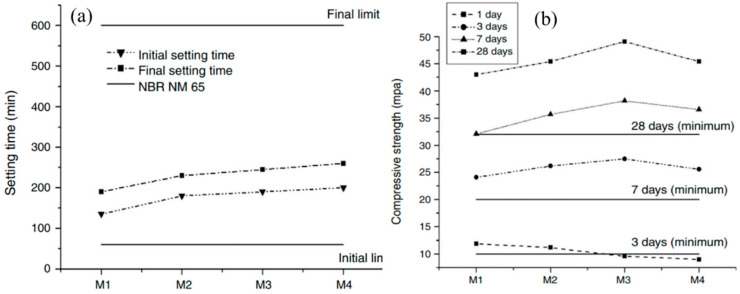
Effect of different ratios on material properties. (**a**) Setting time. (**b**) Compressive strength [43].

The high compositional similarity between DG evil and natural gypsum suggests that DG is a potential replacement for natural gypsum as a cement retarder. Mortar was prepared using 56.5% clinker, 10% limestone, 30% granulated blast furnace slag and DG/natural gypsum [43]. DG: natural gypsum: M1 (0:3.5); M2 (1:2.5); M3 (2.1:1.4); M4 (3.5:0). As shown in Figure 6a, the cement mortar setting time affected the percentage of DG content. The setting time increased with the increase in DG. The mortar produced with only DG took approximately 1 h longer to set than the mortar generated with only natural gypsum. Figure 6b shows the compressive strength experiments, where both DG and natural gypsum were mixed and used together. The compressive strength of the mortar with 2.1% DG and 1.4% natural gypsum was greater than that of the other mixes. The performance of cement prepared by mixing DG and natural gypsum was better than that of cement prepared with pure natural gypsum.

PG has the ability to replace natural gypsum as a cement retarder [60]. In the process of cement hydration, the dissolved SO_4_^2−^ of PG reacts with hydrated calcium aluminates to generate polysulfide-like ettringites, which are adsorbed on the surface of PC clinker particles, which can slow the process of cement hydration and thus achieve retardation. Low-strength gypsum blocks were prepared with PG as the main raw material and light aggregate, filler, fiber reinforcing material, and a foaming agent as auxiliary raw materials. In the absence of other gypsum, PG promoted the growth of the slurry. Moreover, low-carbon and environmentally friendly new wall materials and gypsum blocks were prepared due to their high porosity and low bulk weight; thus, these materials also had other environmental properties of building materials [61].

Cementitious materials were prepared using 74% clinker, 12% cinder, 5% granulated blast furnace slag, 3% limestone, and 6% gypsum. Titanium gypsum, natural gypsum, and DG were compared as cement retarders [51]. The results showed that when titanium gypsum was used as a retarder, the net slurry flow was only 48% of that of the natural gypsum with DG as a retarder specimen, and the setting time was reduced by approximately 70 min. Then, titanium gypsum was treated by mixing titanium gypsum and fly ash 3:1 and activating it under natural conditions; another method was to roast titanium gypsum at 650 °C for 2 h. Then, the flow rate was measured, and it was found that the titanium gypsum after activation or heat treatment was much better than the untreated titanium gypsum in terms of the retardation effect. In the compressive strength experiment, the strength of the cementitious material prepared from treated titanium gypsum reached more than 51 MPa. This shows that pretreatment with titanium gypsum can reduce the use of TG in cement retarders instead of natural gypsum or DG, which greatly enhances the resource utilization of titanium gypsum.

Currently, DG, PG, TG, and CG can act as cement retarders. By replacing natural gypsum as a retarder with DG, PG, and TG, it was found that the dosage of the three types of retarders was approximately 6%. Compared with natural gypsum, the setting time of DG was extended by approximately 1 h, that of PG was increased by 30 min, and that of TG was increased by approximately 2 h. The setting time of TG was similar to that of natural gypsum after treatment with TG. The amount of gypsum citrate used as a cement retarder was only 1.5–3% when the fluidity met the standard.

### 3.3. Road Base Material

The road base material is a layered structure made of a single material on the surface of the roadbed bedding layer in accordance with certain technical measures. The base layer is located directly under the asphalt surface layer with high and medium good materials paving the main load-bearing layer, when the pavement structure is in an important part. The high strength, stiffness, and stability of the grass-roots layer ensure the good quality of the surface structure. At present, all kinds of gypsum in road base materials have been studied.

Thai standard natural road materials were prepared using DG, cement, and lateritic soils, and the samples, maintained for 28 days, were tested for their mechanical properties [5]. It was found that the greater the content of DG, up to 5%, the greater the 28-day UCS. Reducing the amount of cement by adding DG can significantly reduce the cost of road materials.

**Figure 7:**
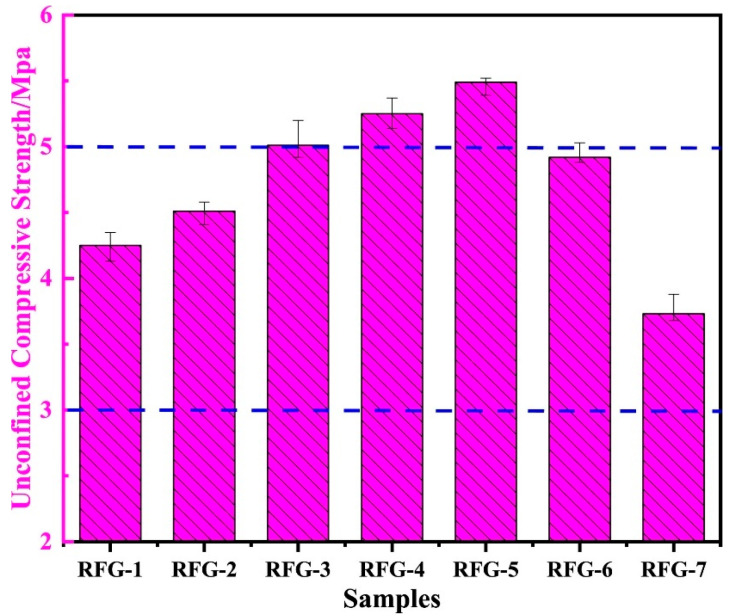
Seven-day unconfined compressive strength of different proportions [62].

Road base materials were prepared using red mud, fly ash, and DG as raw materials. According to the difference in the calcium–silicon ratio, the effect on the mechanical properties of the material was investigated [62]. As shown in Figure 7, from RFG-1 to RFG-7, the calcium–silicon ratio gradually decreased, with ups and downs in strength. At a calcium–silicon ratio = 0.88 (RFG-5), the 7 d unconfined compressive strength (UCS) of the sample reached 5.49 MPa. Compared with red-mud-based road materials, the three kinds of solid waste had synergistic effects and improved the performance of the materials. The hydration products were mainly C-A-S-H gels, with the calcite and zeolite phases dominating.

Currently, there is a high demand for materials in the road-paving process. Three different proportions of PG–lime–fly ash blends were tested for their various performances [47]. The results show that the compressive resilient modulus of the specimens increased slowly with time, but the growth rate decreased, and at the same time, the compressive resilient modulus decreased with the increasing PG content. The UCS of the three specimens met the standard, and at the same time, the increase in the PG decreased the drying shrinkage.

**Figure 8:**
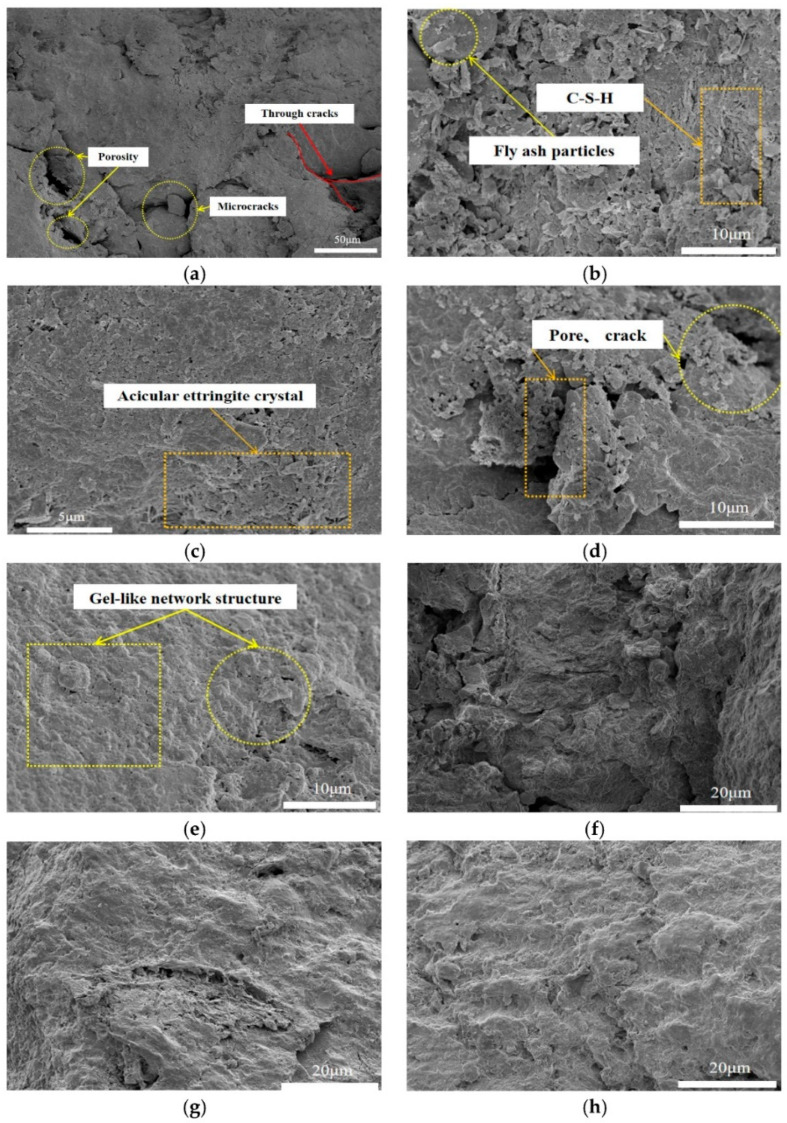
SEM images: (**a**) natural red clay; (**b**) 7 d, 0% PG; (**c**) 7 d, 4% PG; (**d**) 28 d, 0% PG; (**e**) 28 d, 4% PG; (**f**) 60 d, 0% PG; (**g**) 60 d, 4% PG; and (**h**) 90 d, 4% PG [64].

Sarra Meskini prepared new road materials using lime, PG, and fly ash, where the raw material admixture was PG:fly ash:lime = 40:42:18, and the mechanical properties of the resulting road materials met the standards [63]. However, there are harmful impurities in PG. The environmental properties of the material were further investigated, and the addition of fly ash and lime reduced radioactivity by 82% compared to the PG-based road material, while the curing effect of many heavy metal ions was greatly improved. Road base materials were prepared from PG, lime, and fly ash, and the mechanical and expansion properties of roadbed materials with different PG contents and curing ages were tested [64]. Moreover, SEM and XRD were also tested on the sample. The results showed that PG could improve the early strength and stability of the sample, and the mechanical properties increased with increasing age. The temperature stabilized after 60 days. As shown in Figure 8, microscopic analyses revealed that the formation of calcite after the addition of PG increased the compressive strength, while the C-S-H gel effectively filled the soil voids. With increasing strength, the proportion of the gelatinous mesh structure increased, which further increased the strength.

The use of the industrial byproduct gypsum in road base materials is relatively limited. Excessive DG causes the volume of road material to expand, so for road materials, DG production is also basically below 10%, and water glass can be used to reduce the CaSO_4_ content in the system to improve its stability. PG doping is relatively high, up to 15%, and PG can improve the properties of materials.

### 3.4. Filling Material

DGs also have more applications in filling materials. This study investigated environmentally friendly composite filling materials with calcium carbide slag (CCS), fly ash, granulated blast furnace slag, and DG, and used orthogonal tests to study the standard consistency of the water consumption of the raw materials [14], their setting time, and their strength; the results showed that as the DG content increased, the DG content among the raw materials increased. DG had the greatest influence on the standard weekly water consumption and setting time of the system. The optimum performance of the backfill material was achieved when the DG content was 9.1%.

Marvelous Mareya heat-treated DG and mixed it with fly ash to prepare cementless filling materials [65]. The effect of different temperatures and different contents of DG on the properties of the filling materials was investigated. The results showed that the performance of the material reached its maximum value at 30% doping after heat treatment of DG at 60 °C for 2 h. The 28-day UCS was 7.14 MPa. Meanwhile, the material had good durability, which is one of the effective ways to prepare filling materials. Steel slag (SS), granulated blast furnace slag (GBFS), and DG, together with iron ore tailings (IOTs), were the filling materials prepared for construction pits [44]. A new type of filling material was prepared with mechanical activation (by ball milling to change the fineness of the raw material) at a steel slag:granulated blast furnace slag:DG = ratio of 58:32:10, mixed with 79% iron tailings (plus 0.18% water-reducing agent). The experimental slump was 215 mm, and the 28 d compressive strength reached 6.22 MPa. In addition to acting as a retarder in the system, microscopic analyses also confirmed that DG mainly provided the required SO_4_^2−^ in the system to generate ettringite to enhance the strength of the system.

**Figure 9:**
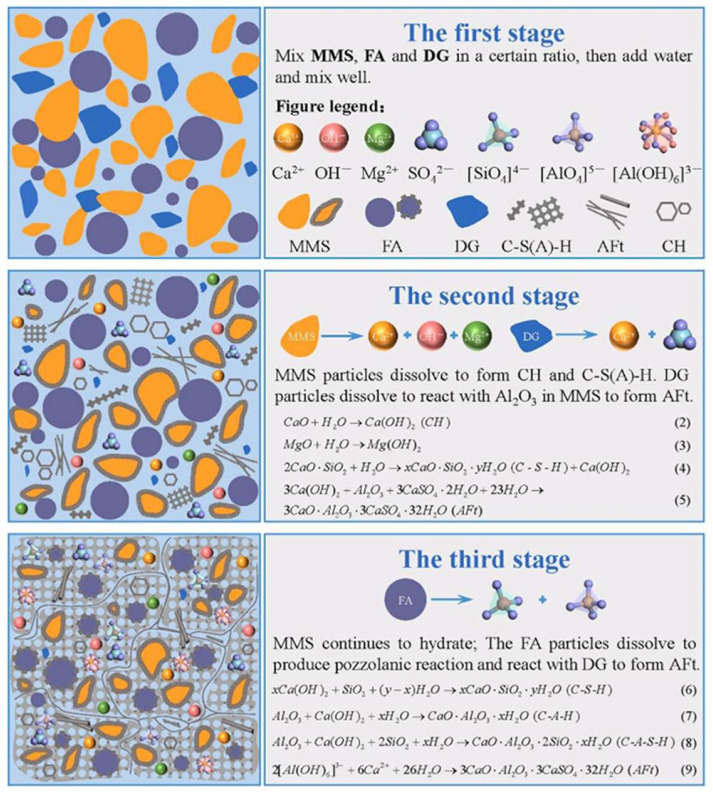
Hydration mechanism diagram [66].

Shishan Ruan developed a new type of filling material using MMS (modified magnesium slag), FA (fly ash), and DG [66]. The results showed that the optimum unconfined compressive strength at 28 days was 5.6–19.30 MPa. According to microanalysis, DGs play a crucial role in the system. In the first stage, DG can promote the dissolution of the two raw materials after contact with water. And then, DG reacts with al in the raw material to produce ettringite. Finally, the gradual increase in the alkalinity promoted the gradual depolymerization of the vitreous reticulation in FA to form [AlO_4_]^5−^ and [SiO_4_]^4−^, as well as polymerization with Ca^2+^, OH^−^, and SO_4_^2−^ to form abundant C-S(A)-H gels and ettringite, as shown in Figure 9.

**Figure 10:**
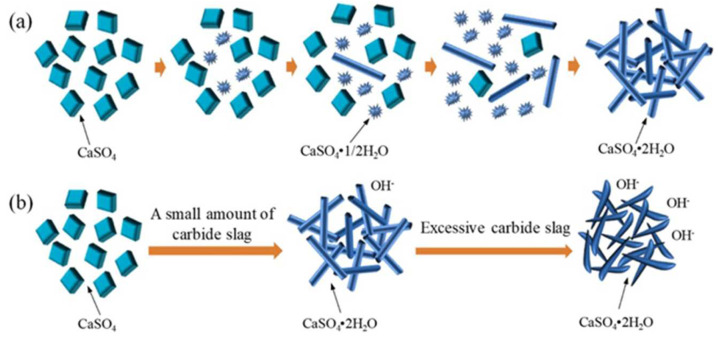
Hydration and hardening mechanism. (**a**) Unblended calcium carbide slag. (**b**) Doping with calcium carbide slag [67].

Xuhong Zhou also investigated the mechanism by which calcium carbide slag stimulates anhydrous PG filling materials [67]. When no calcium carbide slag is added, the hydration and hardening process of anhydrous the PG filling material is extremely slow, as shown in Figure 10a; when gypsum comes into contact with water, some of the gypsum and water molecules form amorphous semi-aqueous gypsum over time. Finally, with the continuous adsorption and reaction of water molecules, columnar dihydrate gypsum is formed. However, the whole reaction process is very slow. The main component of CaO in calcium carbide slag reacts quickly with water to form calcium hydroxide. The OH^−^ provided by calcium hydroxide accelerates the combination of gypsum and water to form hemihydrate gypsum. Subsequently, the hemihydrous gypsum is rapidly transformed into CaSO_4_·2H_2_O. Therefore, the addition of calcium carbide slag accelerates the hydration of the anhydrous PG filling material, as shown in Figure 10b.

Chemical activators such as 40% FG, 25% cement, and 5% granulated blast furnace slag are used as raw materials to formulate a filling material with good curing properties for tailings [68]. The 28 d compressive strength reached 1.44 MPa, and the flow and fluidity of the solidified tailings slurry and mortar are great. FG-based filling materials with 1000 g coal gangue, 300 g fly ash, 300 g FG, 50 g lime, and 78% mass concentration can also be used, and the UCS of the 28-day sample reaches 4–5 MPa [31].

DG and PG also have good applications in filling materials. Filler materials with high solid waste blending were prepared by mixing DG at 10% and PG at about 65%. The role of gypsum in the system was basically the same: SO_4_^2−^ ions were added to the system to generate calomel and C-S-H gels to improve the compressive strength of the filling material. The preparation of highly doped PG as a filling material has great potential, not only because of its high cost, but also because of its positive impact on the treatment of bulk solid waste.

### 3.5. Other Materials

Foam concrete is currently one of the most valuable porous materials in the construction industry due to its low material consumption, low thermal conductivity, low density, lack of need for autoclaving, etc.; it has attracted much attention in the construction industry [69].

DG and recycled water were utilized to partially replace cement with fly ash and blast furnace slag to prepare concrete [70]. It was shown that the addition of DG and recycled water resulted in a partial increase in the compressive strength of the concrete. However, the incorporation of DG should be kept at about 5%, and higher DG will reduce the later strength of concrete. Also, the freezing resistance and carbonation resistance of concrete were improved by adding DG. Mainly, the increase in ettringite in the system and the mesh architecture of C-S-H made the architecture more dense, which had a significant effect on the strength enhancement.

Lightweight porous materials were prepared using DG, NaHCO_3_, and hydroxyethyl methyl cellulose [71]. It was found that by adding hydroxyethyl methyl cellulose, the void fraction of the material was significantly increased. Meanwhile, the addition of NaHCO_3_ reacted to generate CO_2_, which improved the pore size of the material, and NaHCO_3_ also improved the retardation of DG. The lightweight, porous material prepared had a 75% increase in compressive strength as well as reduced thermal conductivity. Antonio Telesca prepared prefabricated building materials using 40% DG, 35% NaOH, and 25% fly ash [72]. It was found that the optimum curing temperature for the materials was 85 °C when the conversion of some of the hydration products in the system was the highest, increasing with the curing time.

**Figure 11:**
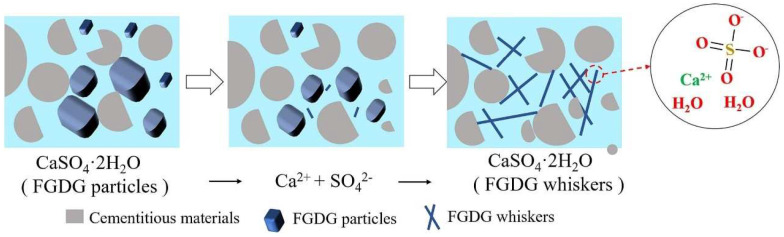
Reinforcement mechanism of DG on the grouting materials [73].

The geopolymer grouting materials were prepared using DG (FGDG), fly ash, and calcium carbide slag [73]. The effects of DG on the material at different temperatures were discussed. The results showed that DG could increase the compressive strength of the material by more than 11% after drying at 120 °C. The hydration reaction of the system was also promoted, with more than 3% more hydration products being generated. The microanalysis revealed that CaSO_4_·2H_2_O in DG mostly had a lumpy form during the initial growth period. As shown in Figure 11, part of the DG was integrated into the slurry, producing a large amount of Ca^2+^ and SO_4_^2−^ with increasing reaction time. The Ca^2+^ and SO_4_^2−^ ions began to disperse and formed needle-like whiskers, but a blocky structure still existed. By the late stage of growth, the blocky mechanism had almost disappeared and the number of needle-like structures gradually increased. This indicates that the drying temperature of DG has a huge impact on the application of DG in materials, and increasing the temperature will promote hydration.

**Figure 12:**
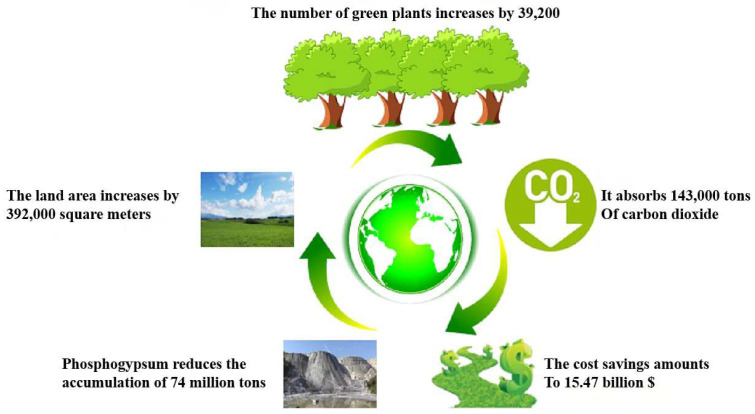
Evaluation of social and economic benefits [76].

To recycle PG for the production of porous concrete, ferrous slag, cement, and hydrogen peroxide were added to prepare concrete [74]. The results of the study showed that hydrogen peroxide and PG had a positive effect on the performance of the system, while the strength of the concrete was up to 7.95 MPa and the density was as low as 830 kg/m^3^, and the final optimum admixture of PG was 5%. HKJHK added PG to natural gypsum to prepare a refractory gypsum board [75]. It was found that gypsum boards prepared by replacing 30% of the natural gypsum with PG increased the efficiency of compressive strength by up to 41.3%. However, the best results in terms of fire resistance and mass loss of gypsum boards were obtained at a substitution rate of 10%. Therefore, PG can be used as a good insulation material for enhancing the fire resistance of materials. Figure 12 shows how Enlai Dong [76] used 40% PG to make a new type of concrete and illustrates its economic benefits under the premise of satisfying various properties. The production of PG-based lightweight concrete consumes 74 billion tons of waste per year, and if planted on these lands, it can indirectly release more than 90,000 tons of oxygen, which greatly improves the environment.

TG was used to prepare cement-based, self-leveling mortar, and the strength, fluidity, etc. [77], of TG were 45%. By adding TG, the swelling property of mortar was reduced to 0.1% (standard is 0.15%). Moreover, XRD, SEM, and thermogravimetry were used to study the microstructure of the samples. CaSO_4_·2H_2_O in titanium gypsum reacted with Al in cement to generate more ettringite, which densified the structure and thus improved the strength.

The industrial byproduct gypsum has also been used in a number of other building materials, but relatively little research has been conducted on this topic. The possibilities for application include various types of concrete blocks, wall materials, and unburnt bricks. The research field of industrial byproduct gypsum should also be expanded, and joint development in multiple fields can realize the recycling of resources.

## 4. Conclusions and Prospects

This review clearly describes the physicochemical properties of various industrial byproducts of gypsum and highlights their potential for application as building materials. The main conclusions of the paper are as follows:

(1) Research has shown that DG is equally suitable for all types of building blocks, with products designed in the appropriate proportions having good physical properties. However, the amount of DG was basically less than 10%. Excessive DG substantially affects the compressive strength and setting time of the slurry, and causes expansion cracking.
(2) PG has also been shown to be a potentially viable construction material, although its low strength and presence of a number of impurities cause it to be underutilized. When PG is used in combination with other solid wastes (PG–red mud systems and PG–fly ash–steel slag), its mechanical properties can be improved. Moreover, compared with DG, PG can be blended up to approximately 30%, which is the main advantage of its industrial reuse.
(3) Other gypsum byproducts have been less investigated, mainly because of the presence of various types of factors. For example, TG has a high Fe content, and impurities in FeSO_4_ lead to decreases in cement strength. The slow hydration rate and low early strength of FG make it difficult to develop and utilize directly, and prevent it from being incorporated into materials at too high a ratio. These impurities reduce the utilization of the byproduct gypsum.

At present, research on the industrial byproduct gypsum in China is constantly improving, and comprehensive utilization methods are constantly expanding. The application scope is extending from the traditional field to the emerging field, and it has gradually formed a scale and industrialization. The waste utilization efficiency is improved, and economic and social development is promoted. However, there are still some urgent problems that need to be solved. Additionally, the particles of the industrial byproduct gypsum are fine, and the development of crystals is poor. It is important to investigate the influence of different technological factors on the growth of industrial gypsum. Improving the performance of industrial byproduct gypsum products by adding modifying materials is the main aspect of current research, but the research and development of modifiers are relatively slow, and the mechanism of their influence on gypsum hydration needs further study.

## Figures and Tables

**Table 2:** Chemical composition of the various types of gypsum.

Species	CaO	Al_2_O_3_	SO_3_	SiO_2_	Fe_2_O_3_	TiO_2_	Refs.
DG	35.92–50.83	1.10–1.16	42.72–47.94	2.01–3.45	0.48–0.64	-	[1,37,38]
PG	32.43–44.92	0.13–0.47	43.05–53.54	2.32–4.13	0.03–0.18	-	[30,32,39]
TG	31.21–35.24	1.12–2.13	27.05–31.16	1.20–2.13	12.02–16.21	1.41–3.83	[24,33,34]
FG	33.62–36.34	0.61–0.72	44.64–53.26	1.17–1.93	0.43–0.82	-	[31,35,36]

**Table 3:** Preparation of building materials from the industrial byproduct gypsum.

Types of Building Materials	Gypsum and Content	The Optimal Proportion	Performances	Refs.
Cementitious material	DG: 6%	26.4% Red mud, 17.6% Fly ash, 50% cement	28 d Compressive Strength: 50.6 MPa	[40]
Cementitious material	DG: 5%	60% Red mud, 24.5% Fly ash, 10.5% Lime	28 d Flexural strengths: 3.2 MPa	[41]
Cementitious material	DG: 9%	64% Circulating fluidized bed fly ash, 27% Carbide slag	28 d Compressive Strength: 6.35 MPa	[42]
Cement retarder	DG: 2.1%	56.5% Clinker, 10% Limestone, 30%Slag, 1.4% Natural gypsum	Extended condensation time 1 h	[43]
Filling material	DG: 9.1%	12.1% Carbide slag, 60.6% Fly ash, 18.2% Granulated blast furnace slag	28 d Compressive Strength: 3.58 MPa	[14]
Filling material	DG: 10%	58% Steel slag, 32% Granulated blast furnace slag	28 d Compressive Strength: 6.22 MPa	[44]
Gypsum plasters	DG: 30%	12% Portland cement	28 d Compressive Strength: 7.21 MPa	[45]
Cementitious material	PG: 5%	20% Red sandstone, 75% Cement	28 d Compressive strength: 62.5 MPa	[46]
Cementitious material	PG: 30%	70% Cement	28 d Compressive strength: 52.1 MPa	[30]
Road base materials	PG: 15%	76% Crushed stone, 12% Fly ash, 6% Lime	28 d Unconfined compressive strength: 4.1 MPa	[47]
Geopolymer concrete	PG: 25%	75% Fly ash, Partial additives	28 d Compressive strength: 51.52 MPa	[48]
Foam concrete	PG: 49%	25% Cement, 20% Fly ash, 6% Hydrated lime	Compressive strength: 1.7 MPa, Dry density: 521.7 kg/m^3^	[39]
Fine-grained concretes	PG: 15%	85% Biomass bottom ash	28 d Compressive strength: 30 MPa	[32]
Cementitious material	TG: 35%	10% Cement, 30% Granulated blast furnace slag, 5% Clinker, 20% Fly ash	28 d Compressive strength: 37.8 MPa	[49]
Cementitious material	TG: 66.5%	20% Cement, 13.5% Microsilica	28 d Compressive strength: 9 MPa	[50]
Cement retarder	650 °C roast TG: 6%	74% Clinker, 5% Granulated blast furnace slag, 3% limestone, 12% Coal cinder	Extended coagulation time: 277 min	[51]
Filling material	FG: 18%	61% Coal gangue, 18% Fly ash, 3% Lime	28 d Compressive strength: 4–5 MPa	[31]
Cementitious material	FG: 40%	55% Granulated blast furnace slag, 5% Cement	28 d Compressive strength: 59.0 MPa	[35]

## Data Availability

Data will be made available upon request.
